# The roles of emergency medical teams in response to Samoa’s 2019 measles outbreak

**DOI:** 10.5365/wpsar.2023.14.6.1031

**Published:** 2024-04-26

**Authors:** Sean T Casey, Natasha A Mamea-Maa, Matilda Nofoaiga, Bronte Martin, Kevin A Henshall, Melissa Fidow, Durgavasini Devanath, Lepaitai B Hansell, Glen Fatupaito

**Affiliations:** a World Health Organization Regional Office for the Western Pacific, Manila, Philippines.; b School of Population Health, Faculty of Medicine and Health, University of New South Wales, Sydney, New South Wales, Australia.; c Ministry of Health, Apia, Samoa.; d Australian Medical Assistance Team, National Critical Care and Trauma Response Centre, Darwin, Northern Territory, Australia.; e New Zealand Medical Assistance Team, Wellington, New Zealand.; f World Health Organization Division of Pacific Technical Support, Suva, Fiji.; g World Health Organization Representative Office for Samoa, American Samoa, Cook Islands, Niue and Tokelau, Apia, Samoa.

## Abstract

**Problem:**

On 15 November 2019, Samoa’s Government declared a state of emergency in response to a rapidly worsening measles outbreak. The outbreak overwhelmed Samoa’s health system, necessitating international assistance, including from emergency medical teams (EMTs).

**Context:**

Measles spread globally throughout 2019, with cases rising by more than 300% in the first quarter of 2019, as compared with 2018. Given Samoa’s low immunization coverage with a measles-containing vaccine at the time, at 40% for the first dose and 28% for the second, the country was soon overwhelmed with measles cases, hospitalizations and deaths.

**Action:**

Following a request for international assistance, 18 EMTs from around the world deployed to Samoa, bringing more than 550 additional clinical, public health and logistics personnel to the country’s measles response. Working alongside Samoan health workers, EMTs provided critical surge assistance in clinical management, vaccination, surveillance, infection prevention and control, risk communication and community engagement, and mental health and psychosocial support.

**Outcome:**

A total of 1867 hospitalized measles patients were treated from 30 September 2019 to 13 January 2020, with 83 measles-related deaths recorded. EMTs provided essential surge support across Samoa’s health system during the most acute phase of the response, helping to care for the ill and control the outbreak.

**Discussion:**

Samoa’s measles response triggered a large-scale and unique EMT activation, with teams integrated into Samoa’s hospitals and health centres. The response demonstrated the critical role that EMTs can play in outbreak response and the importance of strong coordination to ensure optimal use of international clinical surge support by a health system in crisis.

## PROBLEM

On 15 November 2019, Samoa’s Government declared a state of emergency in response to a rapidly worsening measles outbreak, which had initially been declared on 16 October 2019. (*1*) Years of suboptimal coverage of routine immunizations, fuelled in part by both local and international antivaccine campaigners, were compounded by the deaths of two young children following immunization with incorrectly reconstituted vaccines. These deaths and the associated lack of public trust in Samoa’s vaccine programme led to an 8-month suspension of the national immunization programme and contributed to historically low coverage rates, with only 40% coverage of the first dose of measles-containing vaccine (MCV1) and 28% of MCV2. (*2*-*4*) These factors, alongside global and regional increases in measles transmission and reintroduction to Samoa, contributed to the ensuing outbreak and the increased hospitalizations and deaths, overwhelming Samoa’s health system.

## CONTEXT

Measles spread around the world throughout 2019, with global cases rising by more than 300% in the first quarter of 2019, as compared with 2018. (*5*) Significant transmission in the World Health Organization’s Western Pacific Region was observed throughout 2019. (*6*)

Samoa is an independent island nation in the South Pacific, with a population of approximately 200 000 cared for by one tertiary hospital on the main island of Upolu, a referral hospital on the second island of Savai’i, six district hospitals, and a health workforce that includes 98 registered physicians (34 of whom are in private practice), 352 registered nurses, 72 registered midwives, 85 enrolled nurses and 68 allied health professionals. (*7*, *8*) During the 2020–2021 fiscal year, Samoa’s national referral hospital, Tupua Tamasese Meaole (TTM) Hospital (located on Upolu), provided 38 700 outpatient consultations and admitted 1422 patients. (*8*)

**Fig. 1:**
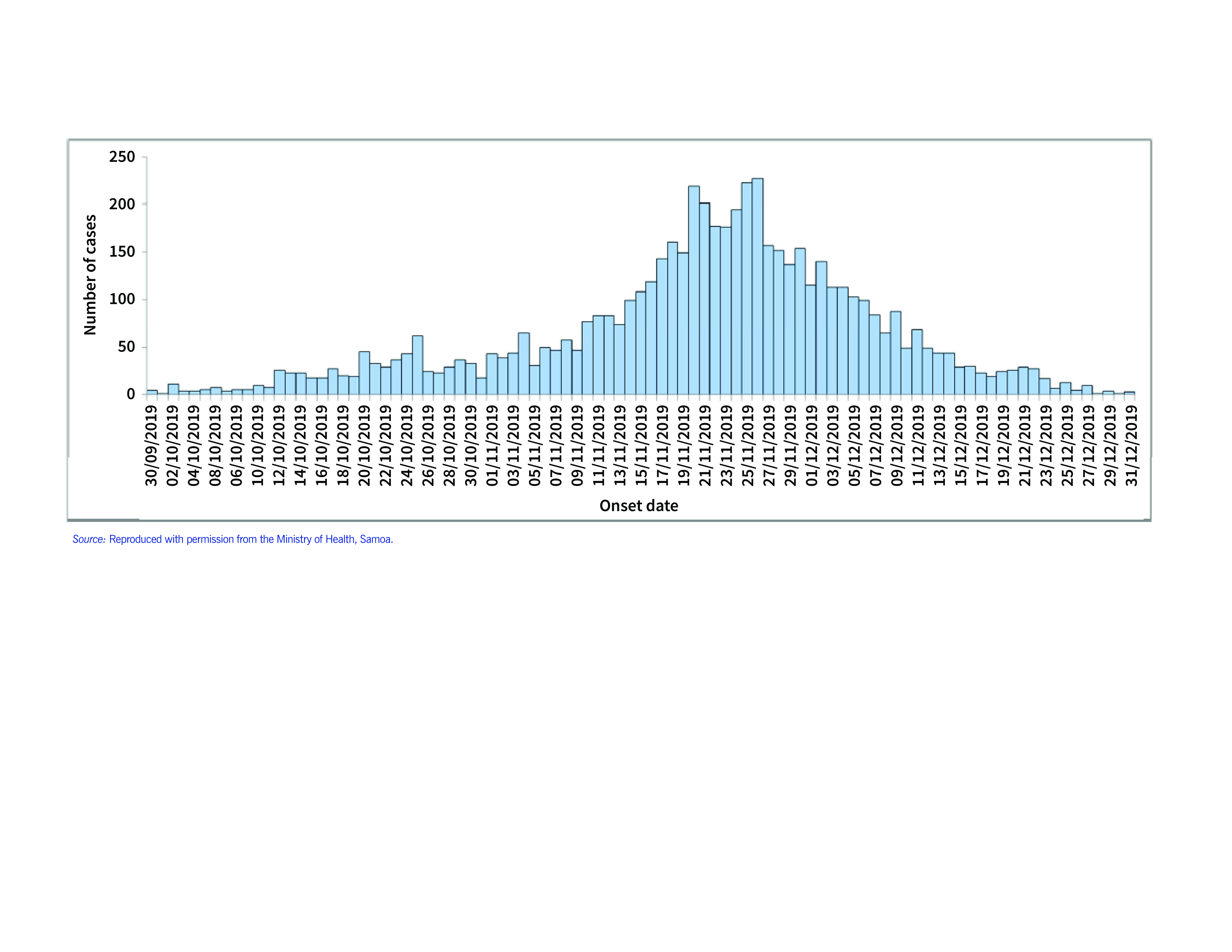
No. of reported measles cases, by onset date, Samoa, 30 September–31 December 2019

Following the introduction of measles into Samoa in late September 2019, a large-scale outbreak ensued, leading to more than 5700 reported cases (only initial cases were confirmed by laboratory testing, with subsequent cases recorded based on clinical diagnosis) (**Fig. 1**). (*2*, *7*) Samoa’s measles outbreak led to a massive surge in paediatric hospitalizations – approximately equivalent to Samoa’s total annual number of hospital admissions occurring from 30 September 2019 to 13 January 2020 – overwhelming national health-care capacity and particularly the country’s sole intensive care unit, which had only six beds, necessitating significant clinical surge support.

[**insert** Figure 1]

## ACTION

In November 2019, recognizing its limited capacity to manage the surge in measles cases, Samoa’s Ministry of Health (health ministry) sought support from the Government of Australia. This led to the deployment of the Australian Medical Assistance Team (AUSMAT), an international emergency medical team (EMT) classified (i.e. having undergone peer verification against global standards) by the World Health Organization (WHO). (*9*) AUSMAT deployed a multidisciplinary team to support Samoa’s measles response, working alongside Samoan health professionals at TTM Hospital to rapidly expand capacities for paediatric critical and emergency care, support clinical coordination, ensure continuity of essential services and provide surge capacities for epidemiology and health informatics. AUSMAT deployed specialized clinicians, tents and equipment to establish 28 additional critical care beds, doubling Samoa’s critical care capacity and creating a dedicated tented high-dependency step-down unit.

AUSMAT’s deployment was soon followed by the New Zealand Medical Assistance Team (NZMAT). NZMAT initially provided clinical medical and nursing support to Leulumoega District Hospital, then supported an expanded second rotation of personnel at Samoa’s Health Emergency Operations Centre as well as primary care services.

Samoa’s health ministry established an EMT Coordination Cell within its Health Emergency Operations Centre, which was jointly led by personnel from the Ministry and WHO, with embedded support from NZMAT. On 27 November 2019, as measles cases and hospitalizations surged, Samoa’s health ministry sought support from WHO to mobilize a larger-scale international EMT response. The WHO global EMT Secretariat published a call for international EMTs on 27 November 2019, requesting specialist teams that were already classified or in the process of completing WHO EMT classification, and which could work in support of Samoa’s national health system. The call requested EMTs with specialist capacity in intensive care, paediatrics, maternal health, emergency medicine, mental health and psychosocial support, infectious diseases, rehabilitation and outpatient care. Teams were asked to submit written offers of support via WHO confirming their compliance with EMT principles and standards as outlined in *Classification and minimum standards for emergency medical teams* (known as the EMT Blue Book), (*10*) their ability to work in English and to be accountable to the local population and the health ministry, to be fully self-sufficient and able to manage their own logistical arrangements, and their commitment to a deployment period of at least 4 weeks.

The call for international EMT support received swift and extensive responses. Within days, international EMT deployments had been offered and accepted by Samoa’s Government, and teams began arriving in Samoa. EMTs arrived from Australia, French Polynesia (France), Hawai’i (United States of America), Israel, Japan, Kiribati, New Zealand, Norway, Papua New Guinea, Solomon Islands and the United Kingdom of Great Britain and Northern Ireland. Several additional teams were deployed by nongovernmental organizations (NGOs) including the Adventist Development and Relief Agency, Doctors without Borders, New Zealand’s Pasifika Medical Association, Save the Children and Samoan Doctors Worldwide. A specialized midwifery team was also mobilized by the United Nations Population Fund (UNFPA).

All EMTs worked within Samoa’s health system, and the EMT Coordination Cell was the primary interface among incoming teams, the health ministry and the Ministry of Foreign Affairs. The EMT Coordination Cell facilitated the acceptance, arrival logistics, clinical tasking and licencing of international EMTs. Every EMT health worker who deployed to Samoa was issued a temporary practice certificate by relevant departments in the health ministry and the Samoa Council of Nursing and Midwifery, facilitated by the EMT Coordination Cell.

Clinical coordination of EMTs was led by the health ministry with support from AUSMAT, NZMAT and WHO. A table summarizing the EMTs that were operational in Samoa, those whose arrival was pending and departing teams was presented in the daily Health Emergency Operations Centre meeting, enabling strategic planning and decision-making. More than 550 personnel from 18 EMTs were deployed throughout the response, including 294 nurses, 156 physicians, 29 logisticians, 23 midwives and 18 allied health professionals, among other technical, public health and support personnel (**Table 1**). TTM Hospital’s Acting Director of Nursing was appointed as the primary clinical liaison at the hospital, integrating EMT clinicians into hospital rotations and coordinating rosters. Collaboration between TTM Hospital’s senior clinicians, specialists from international EMTs and WHO advisers facilitated rapid establishment and revision of national clinical protocols in response to the evolving outbreak.

**Table 1:** Emergency medical teams deployed to Samoa to assist with a measles outbreak, in order of arrival, 2019

Order of arrival	Team	Country of origin	Period of deployment to Samoa	Number of deployed personnel	Scope of engagement
1	Australia Medical Assistance Team^a^	Australia	9 November 2019– 5 January 2020	> 110	Emergency medicine, intensive care medicine, high-dependency paediatrics, infectious diseases, radiology, laboratory specialist assistance, in addition to technical support for water, sanitation, oxygen, biomedical systems, epidemiology and surveillance, health informatics, vaccine data management
2	New Zealand Medical Assistance Team^a^	New Zealand	19 November– 20 December 2019	40	Emergency medicine, intensive care medicine, paediatrics, nursing, midwifery and biomedical systems
3	Counties Manukau Health	New Zealand	20 November– 18 December 2019	60	Intensive care and vaccination
4	New Zealand Red Cross	New Zealand	24 November 2019–1 February 2020	41	Nursing support for surgery, paediatrics, obstetrics and vaccination
5	Doctors without Borders	Australia and France	30 November– 16 December 2019	3	Mobile clinical support for district hospitals; support for the development of clinical management protocols
6	French Polynesia Emergency Medical Team	French Polynesia	30 November–8 December 2019	13	Paediatrics and vaccination support
7	Pasifika Medical Association Medical Assistance Team	New Zealand	2 December 2019–26 January 2020	62	Emergency medicine, intensive care medicine, mental health care, psychosocial support and vaccination support
8	Japan Disaster Relief Infectious Diseases Response Team^a^	Japan	4–28 December 2019	15	Infectious diseases, paediatrics, and hospital and health centre support
9	Hawai’i Health Corps	United States	5–20 December 2019	70	Vaccination support, intensive care medicine
10	Norway emergency medical team^a^	Norway	7 December 2019– 1 January 2020	20	Intensive care, anaesthesia and paramedicine support
11	Save the Children	United Kingdom	7 December 2019–11 January 2020	10	Reproductive and family health care
12	United Kingdom Emergency Medical Team^a^	United Kingdom	7–29 December 2019	31	Intensive care, paediatrics and physiotherapy support
13	Adventist Development and Relief Agency^b^	Australia and New Zealand	9–21 December 2019	7	Intensive care nursing
14	Israel emergency medical team^a^	Israel	9–21 December 2019	11	Intensive care medicine, paediatrics
15	Pacific Community, Kiribati and Solomon Islands teams	Kiribati and Solomon Islands	10–23 December 2019	4	Emergency medicine
16	United Nations Population Fund midwifery team	United Nations Population Fund Pacific Island Countries	18 December 2019– 28 February 2020	10	Reproductive health care and family planning support
17	Papua New Guinea emergency medical team	Papua New Guinea	23 December 2019– 18 January 2020	12	Nursing support for surgery, immunization, and antenatal and paediatric critical care
18	Samoan Doctors Worldwide	New Zealand	22 December 2019–29 March 2020	38	Surgery, paediatrics, intensive care medicine and family medicine support

^a^ These are WHO-classified emergency medical teams.

^b^ The Adventist Development and Relief Agency had a presence in Samoa before the 2019 measles outbreak.

Most EMTs supported the specialist clinical management of infants and children hospitalized with measles. Pasifika Medical Association’s Medical Assistance Team (PACMAT) brought specialist capacity in mental health and psychosocial support, including psychiatrists, psychologists and mental health nurses who aided families affected by measles, staff at the health ministry and EMT members who were caring for the many sick children. Several EMTs also brought clinicians with local cultural understanding, deploying multiple team members from Samoa’s large diaspora community in New Zealand, in particular. Several teams also deployed specialized allied health professionals, including physiotherapists with respiratory expertise and biomedical technicians. EMTs also brought critical equipment, including mechanical ventilators, and supported essential maintenance and logistics related to oxygen provision across hospitals.

In addition to EMTs, other partners including WHO, the United Nations Children’s Fund (UNICEF), the Samoa Red Cross, New Zealand Red Cross and the International Federation of the Red Cross and Red Crescent Societies supported a national vaccination campaign. This campaign included a 2-day national lockdown, with hundreds of local and international health workers travelling across the country in more than 100 integrated mobile teams to vaccinate nearly every Samoan in their home, which ultimately contributed to reduced transmission and the eventual scaling down of EMT operations as hospital admissions declined. (*11*)

## OUTCOMES

Working alongside Samoan health workers, EMTs and other international partners provided critical surge support for case isolation, clinical management, vaccination, epidemiology and surveillance, infection prevention and control, risk communication and community engagement, and mental health and psychosocial support for health workers and affected populations, as well as emergency logistics, nearly doubling the clinical workforce in Samoa’s referral hospital during the most acute phase of the outbreak.

A total of 1867 patients with measles were hospitalized and 83 deaths from measles were recorded. Children aged < 5 years accounted for 2966/5707 (51.9%) cases, 1277/1867 (68.4%) hospitalizations and 73/83 (87.9%) deaths. Most cases, 5332 of 5707 (93.4%), were reported from Samoa’s main island of Upolu. The measles response required an additional

150 inpatient beds at TTM Hospital above the normal bed capacity of 200. At its peak in December 2019, measles cases occupied approximately 185 hospital beds, and there were as many as 21 admissions to intensive care in a single day – more than three times the normal ICU capacity at the hospital.

EMTs helped to expand isolation capacity, supported specialist clinical management and provided essential clinical surge capacity to an overwhelmed health system during the peak of the crisis. In addition to the extensive support provided by international EMTs at TTM Hospital, teams also supported Samoa’s district hospitals and outpatient and primary care facilities. Many teams went beyond direct service delivery to support the overall response by providing training and through skills transfer, and by coordinating support and bolstering critical elements of the response, such as equipment maintenance, oxygen and supply logistics, and information management.

The national vaccination campaign resulted in more than 187 360 children and adults being vaccinated with a measles-containing vaccine in only a few weeks, achieving national vaccination coverage of more than 95%. (*12*, *13*)

Although significantly assisted by international EMTs, the measles response in Samoa was locally led, applying an integrated and unified approach to clinical management across the country’s hospitals and health centres, while leveraging enormous contributions across a wide range of clinical and public health expertise and capabilities from international EMTs, NGOs and other partners.

## Discussion

Samoa’s measles response was the largest EMT mobilization in the Western Pacific Region in 2019. It was a unique EMT activation that included large-scale integration of multiple teams into a small number of hospitals and health centres in the context of an outbreak in a small Pacific island country.

Large-scale EMT mobilizations in 2014 and 2015 were part of the response to outbreaks of Ebola virus disease in West Africa, but these largely led to the establishment of stand-alone Ebola isolation centres. EMTs have also deployed in response to outbreaks of diseases such as cholera, dengue, diphtheria and coronavirus disease (COVID-19).

The response to the 2019 measles outbreak in Samoa demonstrated the potential of EMTs to contribute to such responses and the importance of strong national leadership and coordination to ensure that EMTs are used optimally in support of a national health system in crisis. The activation of EMTs in response to outbreaks has highlighted the need for strengthened mechanisms and standards for these types of deployments; a technical working group on EMT engagement during outbreaks of highly infectious diseases was subsequently formed by WHO’s EMT Secretariat to establish minimum technical and operational standards for these types of response operations.

Integrating EMTs from many different countries – with different ways of working, language abilities, shift patterns, skills, scopes of practice and expectations around care – into a small Pacific island health system proved challenging. Several key enabling factors identified by Samoa’s health ministry and the EMT Coordination Cell included:

the rapid deployment mechanism of the global EMT network that was highly effective in mobilizing capable, self-sufficient teams of specialists within days, with some teams travelling across the globe;
the EMT Coordination Cell’s rapid facilitation of the request for assistance, acceptance of EMTs, EMT deployment, and EMT integration into Samoa’s clinical operations in a short time;
the adaptation of the EMT Coordination Cell’s methodology, particularly in integrating EMTs into local health facilities and adapting reporting approaches to ensure that response actions were locally appropriate and responsive to the country’s needs;
the dedicated Samoan and international emergency coordination and clinical expertise within the EMT Coordination Cell to facilitate strategic planning for the response and day-to-day operational and clinical management;
the pairing of international EMT personnel with Samoan clinicians to facilitate the rapid onboarding, orientation and integration of many foreign clinicians; and
the codesign and rapid communication of measles clinical management guidelines by the health ministry and supporting EMTs to strengthen effective patient management and facilitate the joint work of EMTs and the Samoan health workforce.

Specific challenges related to the EMT activation and how they were addressed included the following:

the majority of teams deployed to Samoa were not WHO-classified EMTs, and some were unfamiliar with EMT methodology and thus needed to adapt in real time;
many EMTs were not specifically designed and trained for outbreak response and had to mobilize additional team members, equipment and supplies that they would not typically deploy, and they had to work in ways that differed from their typical deployments. Expanding EMT rosters to include a wider range of specialists, and developing training and EMT cache (equipment/supplies) to respond to outbreaks may be beneficial to future deployments;
most EMTs are designed to operate their own field hospitals or stand-alone tented clinics, yet the response in Samoa required that they operate within the country’s established hospitals and health centres, necessitating adaptation of some teams’ standard operating procedures;
the standard EMT minimum data set collection system and forms were not optimal, as nearly all affected patients were treated in Samoan hospitals and health centres, rather than in stand-alone EMT field hospitals. Instead of employing the standard EMT reporting mechanisms, each team provided brief updates during regular EMT Coordination Cell meetings and shared detailed exit reports at the end of their deployments. Daily communication was maintained between the EMT Coordination Cell and EMTs through messenger applications and e-mail. These mechanisms were helpful in addressing challenges as they arose and did not create an excessive reporting burden on EMTs; however, they limited the quality and completeness of the data collected. WHO’s EMT Secretariat and the global EMT network should consider how clinical activity reporting can be optimized in integrated deployment settings such as this one;
the large number of incoming teams and personnel within a short time span posed challenges, particularly for Samoan clinical leaders, in terms of integrating them into an already overwhelmed health system. The EMT Coordination Cell adopted several strategies, including semistructured induction briefings, coordination through messenger groups and consolidation of mini-biographies of incoming team members to help smooth this process. EMTs were specifically asked to follow the guidance and direction of Samoa’s clinical coordination personnel to avoid confusion or conflict, and this was largely respected by the deployed personnel; and
some EMT personnel had limited English fluency, which was required for effective clinical practice in Samoa.

Building on lessons learned from the 2019 measles outbreak and the subsequent COVID-19 pandemic response, Samoa’s health ministry formally launched its own national EMT in 2022: the Samoa Emergency Medical Assistance Team, or SEMAT. (*14*) SEMAT will contribute to Samoa being better prepared to respond to health emergencies in the future with its own deployable clinical surge capacity. SEMAT is one of many Pacific nation EMTs established in recent years, emphasizing the growing recognition of the importance of deployable, resourced, trained and coordinated teams to provide clinical surge capacity for the wide range of hazards facing Pacific island countries and areas. (*15*)
